# Bedside pharmacist interventions for managing intravenous drug incompatibilities in critically ill patients

**DOI:** 10.1016/j.rcsop.2026.100863

**Published:** 2026-10-15

**Authors:** Allan Carneiro de Souza, Joiciane Dias Rodrigues Neves, André Luis Félix da Silva Terres, Gabriel Gonçalves da Silva, Luana Ferreira de Almeida, Arnaldo Couto, Sabrina Calil Elias, Selma Rodrigues de Castilho

**Affiliations:** a State University of Rio de Janeiro, Pedro Ernesto University Hospital, Pharmacy Service, Brazil; b Fluminense Federal University, Postgraduate Program in Pharmaceutical Care Management (PPG-GAFAR), Niterói, Brazil; c State University of Rio de Janeiro, Faculty of Nursing, Brazil; d State University of Rio de Janeiro, Pedro Ernesto University Hospital, Patient Safety Center, Brazil; e State University of Rio de Janeiro, Faculty of Biological Sciences and Health, Department of Pharmacy, Brazil; f Fluminense Federal University, Faculty of Pharmacy, Niterói, Brazil

**Keywords:** Patient safety, Drug incompatibility, Medication errors, Administration, Intravenous, Pharmacy service, Hospital, Pharmacists

## Abstract

**Background:**

Patients admitted to intensive care units are at increased risk of intravenous drug incompatibilities, owing to polypharmacy and the limited number of available venous access devices.

**Objective:**

To evaluate bedside pharmacist interventions for managing drug incompatibilities identified during intravenous administration in intensive care unit patients.

**Methods:**

A cross-sectional study with prospective data collection was conducted between June and December 2025 in five intensive care units of a university hospital. Two pharmacists assessed medication administration at the bedside and checked compatibility using the Micromedex and American Society of Health-System Pharmacists (ASHP) Injectable Drug Information databases. The type and number of central venous access devices, infusion modality, proposed interventions, and the healthcare team's response were recorded. Data were described using absolute and relative frequencies.

**Results:**

A total of 887 infusions were analyzed in 88 patients, of which 159 corresponded to occurrences of incompatibility or lack of compatibility data (73 distinct drug combinations). Of these 159 occurrences, 135 (84.9%) were incompatible, and 24 (15.1%) lacked compatibility studies. Most of the pharmacist interventions performed were accepted by the healthcare team (130/159; 81.8%).

**Conclusions:**

The high frequency of incompatibilities observed is related to the large number of intravenous medications and the complexity of therapeutic regimens used in intensive care. Bedside pharmacist involvement proved feasible and contributed to safer medication administration practices in these units.

## Highlights

• Bedside pharmacists directly observed IV drug compatibility in five ICUs.
• Incompatibilities or untested pairs occurred in 70% of patients assessed.
• 159 occurrences were found across 887 infusions in 88 ICU patients.
• 81.8% of pharmacist recommendations were accepted by the care team.
• Findings support integrating pharmacists into bedside infusion checks.

## Introduction

Drug incompatibility is defined as a physical and/or chemical reaction between two or more substances when present in the same solution, which may undermine the safety and efficacy of treatment. These interactions might be classified as physical or chemical and may produce visible signs, such as precipitation, turbidity, color change, or gas release, or may be imperceptible.^1^ Even so, their consequences can be clinically relevant, including loss of drug activity, catheter obstruction, and adverse events of variable severity.1., 2. From a chemical standpoint, these reactions may involve oxidation, reduction, hydrolysis, and the formation of insoluble complexes, resulting in drug inactivation or the formation of potentially toxic by-products.^3^

In the critically ill patient, the high number of intravenous medications combined with limited venous access availability frequently necessitates simultaneous administration via the same line, whether via Y-site connections or multi-lumen devices. This is a practically unavoidable configuration in routine care, which promotes contact between different solutions and consequently increases the risk of incompatibility. For this reason, planning infusion lines and consulting specific compatibility sources in advance are necessary steps in care.^4^

The clinical impact of a drug incompatibility varies according to the drug pair involved, the conditions of preparation and administration, and individual patient vulnerability.^4^ Precipitates and particles formed in these reactions can compromise catheter patency, favoring local complications such as phlebitis and line obstruction, or systemic complications such as embolism and allergic reactions.^5^ Even so, establishing a direct relationship between incompatibility and patient clinical outcomes remains a methodological challenge, since studies conducted under real-world care conditions often cannot systematically observe or measure this clinical implication.^5^

In intensive care units, the combination of intravenous polypharmacy, continuous and intermittent infusions, and a limited number of lumens increases the likelihood of incompatible combinations forming, or combinations for which insufficient compatibility data exist. Identifying and managing these situations forms part of patient safety strategies in this setting.4., 6., 7.

In this context, the clinical pharmacist plays a strategic role in assessing drug infusions, noticing potential incompatibilities, and proposing adjustments to the infusion regimen. Their involvement helps ensure therapeutic efficacy, reduce the risk of serious adverse events, and promote patient safety.^8^

Most studies on intravenous incompatibilities estimate potential incompatibilities from prescriptions, medical records, or electronic records.1., 2., 9., 10., 11. This approach is useful for risk screening but has an important limitation: the coexistence of medications in a prescription does not guarantee that they were actually administered at the same time and via the same line. Direct bedside observation constitutes a more appropriate strategy for recording the patient's actual exposure, taking into account the arrangement of venous access devices, the number of lumens, the infusion modality, and the simultaneity of administration.

Thus, the present study aimed to evaluate bedside pharmacist interventions for managing drug incompatibilities identified during intravenous administration in patients admitted to intensive care units.

## Methods

### Study design, setting, and sample

This was a cross-sectional study with prospective data collection, conducted between June and December 2025 in five intensive care units of a university hospital (neonatal, pediatric, adult, cardiac and surgical), which together comprise 52 beds. Given the descriptive and exploratory nature of the study and the operational infeasibility of assessing Y-site compatibility for all eligible patients simultaneously across the five units, a convenience sample was used. Of the 416 admissions recorded during the study period, 88 patients (21.2%) received an in-person bedside assessment, according to pharmacist availability. Patients with a length of stay of less than 48 h and those prescribed fewer than three injectable medications were excluded.

The sample size was calculated a posteriori, solely to contextualize the sample obtained, and did not guide the recruitment process. Taking as reference the population of patients admitted to the five intensive care units during the study period (*N* = 416), the formula for proportion estimation with finite population correction was used, considering a 95% confidence level (Z = 1.96) and an expected prevalence of Y-site incompatibility of 50%, given the absence of a prior estimate in the local literature. This calculation indicated a minimum sample of 79 patients for a margin of error of 10%. The sample actually studied comprised 88 patients, corresponding to a margin of error of approximately 9.3%, considered adequate for the study objectives.

### Operational definitions

Y-site administration refers to the simultaneous infusion of two or more medications through the same lumen or venous access line, connected by a Y-shaped device; for medications to be administered this way, they must be at least physically compatible.^12^ Multi-lumen catheters (double- or triple-lumen) allow simultaneous administration of different medications through physically independent pathways within the same device, reducing, but not eliminating, the need for concurrent administration via the same line.4., 12. Intermittent infusion refers to administration of a medication as a bolus or over a defined period, followed by line flushing, whereas continuous infusion refers to uninterrupted administration over a prolonged period, without interruption for flushing between doses. Line flushing consists of passing a compatible solution between the administration of two incompatible medications via the same line, to prevent direct contact between them.^12^

Each intravenous medication in use by the patient at the time of the bedside assessment was counted as one infusion, regardless of how many times it was administered over the day. The total of 887 infusions therefore corresponds to the number of intravenous medications assessed across the 88 patients, with a mean of 10.1 medications per patient.

### Data collection

The data collection form was built by the research team based on the study design and the prior definition of the variables of interest (unit of admission, date of birth, sex, number and type of central venous access, presence of peripheral venous access, intravenous medications administered via each line, incompatibilities identified, description of the intervention, acceptance or not by the healthcare team, and the pharmacist responsible for the assessment).

The two pharmacists responsible for data collection were staff members of the hospital's clinical pharmacy service, with prior experience in intensive care and direct practice in the units evaluated.

Evaluator training was coordinated by the corresponding author, who holds a master's degree in Pharmaceutical Care and is a specialist in Clinical Pharmacy and in Hospital and Clinical Pharmacy. The two pharmacists took part in two training sessions, each lasting three hours, on the use of the data collection instrument and the operational definitions of the variables.

After training, both independently assessed the same six patients in a pilot stage. Agreement between the researchers was assessed regarding the type and number of intravenous incompatibilities identified, the number of venous accesses and lumens, the number of medications prescribed intravenously, and the type of pharmacist intervention proposed.

There was full agreement between the researchers in five of the six patients assessed and partial agreement in one patient. The discrepancies identified were discussed between the researchers, and data collection criteria were standardized before the start of definitive data collection. Acceptance of pharmacist interventions was not assessed during the pilot stage.

Data were recorded on a structured form, including the medications present in each infusion line, peripheral and central venous access devices, the number of lumens, and the mode of administration. Patient characterization variables (age, sex, unit of admission, and length of stay up to the time of assessment) were obtained from a report generated by the institutional electronic health record system. The bedside assessment took place once during each patient's admission.

An observed incompatibility was defined as the concomitant administration, via the same line, of medications classified as incompatible in the sources consulted. Pairs classified as not tested, that is, lacking available compatibility data, were recorded separately and, like incompatible pairs, were the target of pharmacist intervention, forming part of the primary intervention outcome.

Drug incompatibilities were verified by consulting specialized databases widely recognized in clinical practice, namely Micromedex and American Society of Health-System Pharmacists (ASHP) Injectable Drug Information. When the two sources differed, the more restrictive classification prevailed, following the hierarchy: incompatible over variable, variable over compatible, and incompatible over compatible.

In practice, a combination was classified as incompatible whenever at least one of the two sources classified it as such, including when the other source had no compatibility data for the pair (a “not tested” classification). For example, when Micromedex classified a pair as incompatible and ASHP Injectable Drug Information had no data on that pair, the incompatible classification prevailed. A combination was classified as compatible only when both sources agreed on that classification. This choice of the more conservative classification aimed to prioritize patient safety in the event of disagreement or missing data between the sources consulted.

To make this classification transparent, Table 2 and Supplementary Table S1 present, for each combination identified, the classification and the type of incompatibility individually attributed by each source consulted (Micromedex and ASHP Injectable Drug Information), together with the final classification resulting from the application of this hierarchy, that is, the more restrictive classification between the two sources.

When the classification of one or both sources was variable, an additional tie-break rule was applied: the actual administration conditions used in this study (route of administration, diluent, and infusion mode) were checked against the scenario for which the source indicated incompatibility. When they matched, the combination was reclassified as incompatible, with an explanatory note in Table 2 or Supplementary Table S1, as applicable.

Of the 73 combinations identified, Micromedex and ASHP Injectable Drug Information assigned the same category in 33 of them. In the remaining 40, the two sources diverged as to the category assigned to the pair; in most of these cases (37 combinations), this disagreement stemmed solely from the absence of data in one of the sources (a “not tested” classification), rather than from a conflicting risk judgment, with the category from the source that had data prevailing directly. A substantive divergence, that is, involving genuinely different compatibility assessments between the two sources, occurred in six combinations. For the pairs dexmedetomidine and ketamine (variable by Micromedex and incompatible by ASHP) and potassium phosphate and calcium gluconate (incompatible by Micromedex and variable by ASHP), the more restrictive category, incompatible, prevailed directly, according to the hierarchy adopted. In the other four combinations (ceftriaxone and vancomycin, dexmedetomidine and rocuronium, ampicillin/sulbactam and polymyxin B, and liposomal amphotericin B and meropenem), at least one source classified the pair as variable; in these cases, application of the tie-break rule, based on the actual administration conditions observed in the study, resulted in a final classification of incompatible, as detailed in the notes to Table 2 and Supplementary Table S1.

According to Micromedex, compatibility between medications is classified into four categories: compatible, incompatible, variable, and not tested. The compatible classification indicates that medications can be administered concomitantly without notable physicochemical changes. The incompatible category refers to changes that may compromise the stability, safety, or efficacy of concurrent administration. The variable classification indicates that compatibility is contingent on factors such as concentration, diluent, and contact time, while not tested indicates a lack of sufficient studies and evidence to determine compatibility between the medications.^13^ ASHP Injectable Drug Information classifies drug pairs as compatible, incompatible, or of uncertain outcome, based on the physicochemical and stability standards observed in the studies consulted.^14^

The type and number of central and peripheral venous access devices present at the time of assessment were recorded; for central catheters, the number of devices and lumens (single-lumen, double-lumen, or triple-lumen) were also recorded. This information was obtained from medical records and confirmed with the nursing team at the bedside.

This information was collected because it directly determines the feasibility of administering incompatible medications through physically separate routes: the greater the number of available lumens, the greater the possibility of avoiding concomitant administration via the same line without the need for interventions such as line flushing, staggered administration times, or placement of a new access.

Pharmacist interventions took place whenever concomitant administration of incompatible medications, or medications lacking compatibility data, was identified. For intermittent infusions via the same line, the recommended approach was to flush the system with 0.9% sodium chloride solution, or another compatible solution, and stagger administration times; where appropriate, changing the line for one of the medications was also recommended. For continuous infusions, a separate line was recommended or, when necessary, placement of a new venous access.

Placement of a new venous access was specifically recommended when all lumens and routes already available in the patient were occupied by other continuous infusions or by medications incompatible with one another, leaving no physically separate route to safely administer the medication in question.

Pharmacist interventions were directed mainly at nursing staff and, in some cases, at physicians, when a new catheter was required or when the intervention took place during the multidisciplinary ward round.

All interventions were recorded and classified as accepted or not accepted, with an intervention considered accepted when the healthcare team followed and effectively implemented the recommendation.

### Statistical analysis

Variables were described using absolute and relative frequencies and measures of central tendency and dispersion in Microsoft Excel. No missing or incomplete data were found among the variables assessed. To investigate possible factors associated with the occurrence of incompatibility, the association between unit of admission and occurrence of incompatibility was assessed using the chi-square test of independence, and the difference in the number of central venous access lumens between patients with and without occurrence of incompatibility was assessed using the Mann-Whitney test; both analyses were performed in Python (pandas and SciPy libraries), with significance set at *p* < 0.05.

For the calculation of sampling precision in a finite population, the expression n = [N·Z^2^·p(1 − p)] / [Z^2^·p(1 − p) + N·e^2^] was used, where N represents the population of eligible admissions, p the expected prevalence, Z the critical value corresponding to the 95% confidence level, and e the absolute margin of error.

This comparatively wide margin of error should be considered when interpreting the frequency estimates presented in this study.

### Ethical aspects

The institution's Research Ethics Committee approved the project (CAAE No. 83646024.0.0000.5259; Approval No. 7.175.924). The waiver of informed consent is grounded in the fact that the pharmacist interventions evaluated correspond to the clinical pharmacy service's routine care practice, neither introduced nor modified for research purposes, with the healthcare team retaining full autonomy to accept or decline them. The study was limited to the observational recording of this practice, posing no additional risk to patients and using anonymized data, in accordance with Resolution No. 466/2012 of the Brazilian National Health Council.^15^ This study followed the STROBE (Strengthening the Reporting of Observational Studies in Epidemiology) reporting guideline for cross-sectional studies.

## Results

Of the 416 patients admitted to the five ICUs during the study period, 88 (21.2%) were assessed at the bedside, each observed at a single point during their admission; of these, 62 (70.5%) presented at least one occurrence of incompatibility or of missing compatibility data, totaling 159 occurrences identified (Fig. 1).

**Fig. 1:**
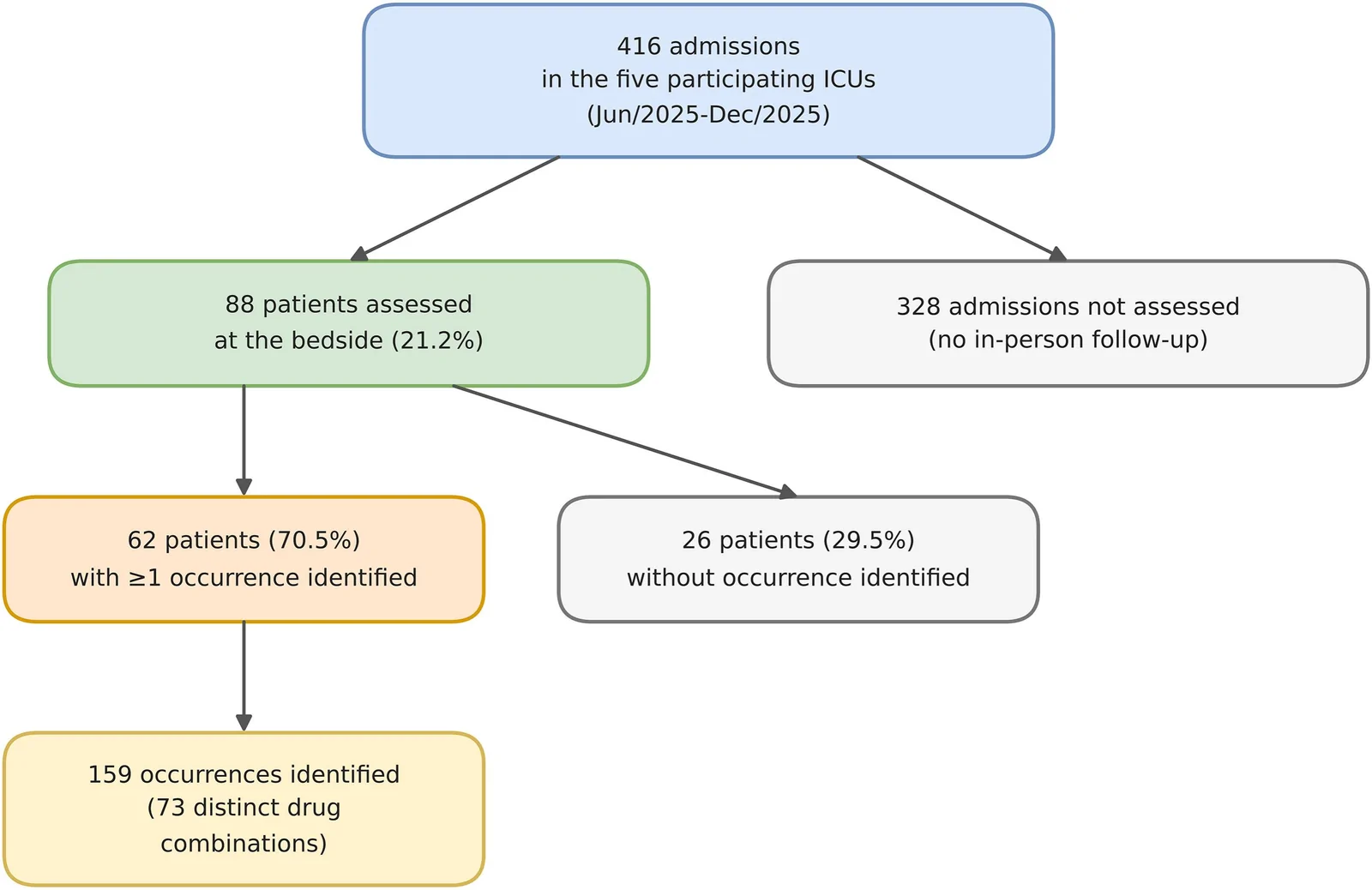
Flow of patient selection and of the occurrences identified at the bedside. Of the 416 admissions recorded in the five intensive care units during the study period, 88 patients (21.2%) were assessed at the bedside; among these, 62 (70.5%) presented at least one occurrence of drug incompatibility or of a pair lacking compatibility data, totaling 159 occurrences involving 73 distinct drug combinations. *Source: the authors.*

Table 1 describes the general characteristics of the sample, including demographic characteristics (age and sex), distribution by intensive care unit, the total number of medications assessed and the mean per patient, as well as the central venous access devices identified.

**Table 1:** General characteristics of the sample and of the central venous access devices.

Characteristic	Result
Patients assessed, n (% of admissions in the period)	88 (21.2%)
Age, years (mean ± SD)	35.6 ± 30.5
Male sex, n (%)	45 (51.1%)
Female sex, n (%)	43 (48.9%)
Length of stay until assessment, days (median, IQR)	47 (24–96)

**Distribution by unit, n**	
Surgical ICU	21 (23.9%)
Adult ICU	19 (21.6%)
Neonatal ICU	18 (20.5%)
Pediatric ICU	17 (19.3%)
Cardiac ICU	13 (14.8%)
Intravenous medications assessed, n (mean/patient)	887 (10.1)
Patients with at least one occurrence of incompatibility (including untested combinations), n (%)	62 (70.5%)

**Central venous access devices, n**	
Triple-lumen catheter	56 (63.6%)
Double-lumen catheter	23 (26.1%)
Two double-lumen catheters	5 (5.7%)
Two catheters (one double-, one triple-lumen)	3 (3.4%)
Single-lumen catheter	1 (1.1%)

Length of stay showed a skewed distribution, with a median of 47 days (interquartile range 24–96 days); for this reason, the median and interquartile range are reported instead of the mean and standard deviation. The longest stays were concentrated mainly in the neonatal and pediatric units, where prolonged admissions are clinically expected.

In the additional exploratory analysis, there was no statistically significant association between unit of admission and occurrence of incompatibility (chi-square test of independence: χ^2^ = 5.65; df = 4; *p* = 0.227). In contrast, the number of central venous access lumens was significantly higher among patients with an identified occurrence of incompatibility than among those without (Mann-Whitney test: *p* = 0.028).

A total of 159 occurrences of incompatible or untested drugs were identified, corresponding to 73 distinct combinations. Among these occurrences, 135 (84.9%) were incompatible, and 24 (15.1%) had not previously been assessed for compatibility. Of these occurrences, 125 (78.6%) were administered intermittently, and 34 (21.4%) included at least one medication given by continuous infusion.

The most frequent combinations, classified as incompatible, involved dexmedetomidine and ketamine, omeprazole and vancomycin, and furosemide and ondansetron. Table 2 presents the 25 combinations identified in two or more occurrences (111 of 159 occurrences, 69.8%); the remaining 48 combinations, each identified in a single occurrence (48 of 159 occurrences), are listed in Supplementary Table S1, provided as a separate file. For each combination, Table 2 and Supplementary Table S1 present the classification and type of incompatibility according to Micromedex and according to ASHP Injectable Drug Information separately, together with the final classification resulting from the hierarchy described in the Methods; when the prevailing classification corresponded to variable in one or both sources, the final classification was determined from the actual administration conditions observed in the study, as indicated in a note in the tables.

**Table 2:** Combinations of incompatible drugs or drugs lacking compatibility data identified in two or more occurrences (*n* ≥ 2).

Drug pair	n (%)	Micromedex classification	Type of incompatibility (Micromedex)	ASHP classification	Type of incompatibility (ASHP)	Final classification
Amikacin x sulfamethoxazole and trimethoprim	2 (1.3)	Incompatible	Physical (turbidity/precipitation)	Not tested	–	Incompatible
Amphotericin B x vancomycin	2 (1.3)	Incompatible	Physical (precipitation)	Not tested	–	Incompatible
Sodium bicarbonate x ondansetron	3 (1.9)	Incompatible	Physical (turbidity, precipitate, color change)	Incompatible	Physical (precipitation)	Incompatible
Sodium bicarbonate x sulfamethoxazole and trimethoprim	2 (1.3)	Incompatible	Physical (turbidity/precipitation)	Not tested	–	Incompatible
Sodium bicarbonate x calcium gluconate	2 (1.3)	Incompatible	Physical (turbidity, precipitate, color change)	Not tested	–	Incompatible
Cefepime x ondansetron	2 (1.3)	Incompatible	Physical (turbidity)	Not tested	–	Incompatible
Ceftriaxone x calcium gluconate	2 (1.3)	Incompatible	Physical (turbidity, precipitate, color change)	Not tested	–	Incompatible
Ceftriaxone x vancomycin ᵃ	2 (1.3)	Variable	Physical (precipitate)	Variable	Not determined	Incompatible
Ketamine x fentanyl	8 (5.0)	Not tested	–	Not tested	–	Not tested
Ketamine x rocuronium	5 (3.1)	Incompatible	Physical (particles)	Incompatible	Physical (microparticles)	Incompatible
Ketamine x furosemide	2 (1.3)	Incompatible	Physical (precipitation)	Not tested	–	Incompatible
Dexamethasone x phenytoin	2 (1.3)	Incompatible	Physical (precipitation)	Not tested	–	Incompatible
Dexmedetomidine x ketamine	16 (10.1)	Variable	Physical (particles)	Incompatible	Physical (microparticles)	Incompatible
Dexmedetomidine x rocuronium ᵇ	3 (1.9)	Variable	Physical (particles)	Variable	Not determined	Incompatible
Phenytoin x meropenem	4 (2.5)	Incompatible	Physical (opacity)	Incompatible	Physical (opacity)	Incompatible
Phenytoin x metoclopramide	2 (1.3)	Incompatible	Physical (precipitation)	Not tested	–	Incompatible
Phenytoin x ondansetron	3 (1.9)	Incompatible	Physical (precipitation)	Not tested	–	Incompatible
Fluconazole x sulfamethoxazole and trimethoprim	3 (1.9)	Incompatible	Physical (turbidity, precipitate)	Incompatible	Physical (viscosity)	Incompatible
Furosemide x ondansetron	13 (8.2)	Incompatible	Physical (turbidity, precipitate)	Incompatible	Physical (precipitation)	Incompatible
Furosemide x vancomycin	8 (5.0)	Incompatible	Physical (turbidity, particles)	Incompatible	Physical	Incompatible
Furosemide x rituximab	2 (1.3)	Incompatible	Physical (microprecipitation, filaments)	Not tested	–	Incompatible
Furosemide x thiamine	2 (1.3)	Incompatible	Physical (turbidity, precipitate, color change)	Not tested	–	Incompatible
Omeprazole x vancomycin	15 (9.4)	Incompatible	Physical (precipitation, color change)	Not tested	–	Incompatible
Ondansetron x sulfamethoxazole and trimethoprim	4 (2.5)	Incompatible	Physical (turbidity, precipitate)	Not tested	–	Incompatible
Polymyxin B x sulfamethoxazole and trimethoprim	2 (1.3)	Incompatible	Physical (turbidity, precipitate)	Not tested	–	Incompatible

ᵃCeftriaxone x vancomycin: variable classification in both sources; considered incompatible under the administration conditions observed in this study.

ᵇDexmedetomidine x rocuronium: variable classification in both sources; considered incompatible under the administration conditions observed in this study.

In total, 159 pharmacist interventions were performed: 106 (66.7%) recommended flushing the access with a compatible solution and staggering administration times, of which 106 (100%) were accepted; 45 (28.3%) involved changing the infusion line, of which 23 (51.1%) were accepted; and 8 (5.0%) involved placing a new venous access, of which only 1 (12.5%) was accepted. Overall, 130 of the 159 interventions (81.8%) were accepted by the healthcare team (Fig. 2).

**Fig. 2:**
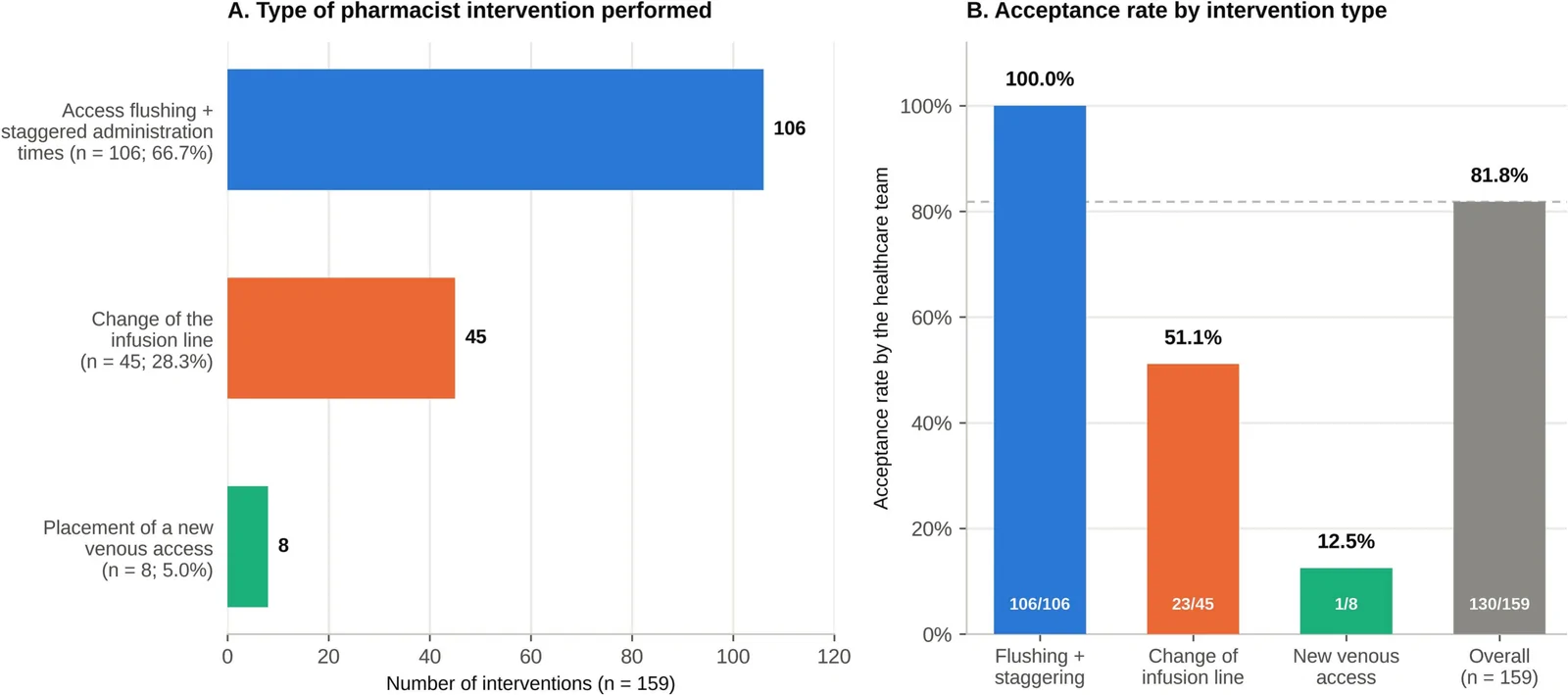
Pharmacist interventions performed for occurrences of incompatible drugs or drugs lacking compatibility data. (A) Type of pharmacist intervention performed (*n* = 159): line flushing combined with staggered administration times, change of the infusion line, and placement of a new venous access. (B) Acceptance of the interventions by the healthcare team, shown by intervention type and overall (*n* = 159). *Source: the authors.* Of the 159 interventions proposed, 29 (18.2%) were not accepted by the healthcare team, distributed across 18 patients.

## Discussion

Direct bedside observation identified concomitant administration of incompatible medications, or medications lacking compatibility data, in most patients assessed, and the healthcare team accepted the pharmacist interventions proposed at the time of administration in most cases. This frequency should be interpreted in light of the method used. Marsilio et al.^10^ reported incompatibilities in 68% of prescriptions in an adult ICU, while Kayra et al.^11^ identified 73.75% in the daily medication lists of another unit. More discrepant frequencies were reported in pediatric (83.3%)^2^ and neonatal (46.3%)^9^ units, the latter considerably lower. All of these studies were based on prescription analyses, with the neonatal study also incorporating medical record data.

In a retrospective study conducted in a historical cohort of adults in critical care units in Brazil, based on the nursing schedule, 565 pairs of medications administered simultaneously were analyzed: 44.9% were compatible and 8.8% potentially incompatible, with a potential incompatibility incidence of 2.7%, predominantly involving combinations of alkaline-pH drugs and weak acids, which favor precipitation in the same line.^16^ These studies are valuable for risk screening but do not confirm simultaneous contact between medications in the same line.

Prospective studies with direct observation have also been conducted in intensive care units. Machotka et al.^17^ followed 82 patients in two ICUs (medical and surgical) of a university hospital, verifying concomitant administration of medications only during periods of higher care workload, and identified incompatibility in 6.82% and 2.16% of the drug pairs assessed in the two units, respectively. Unlike that study, the analysis in the present work was conducted at the patient level, allowing quantification of the proportion actually exposed to at least one incompatibility during the observation period.

This finding is noteworthy when compared with that of Machotka et al.^17^: in the additional exploratory analysis performed in this study, the number of lumens was significantly higher among patients with an identified occurrence of incompatibility, whereas Machotka et al. observed an association in the opposite direction at the unit level, with a lower mean number of lumens per patient in the medical ICU (1.88), associated with a higher incompatibility frequency (6.82%), and a higher mean number in the surgical ICU (2.41), associated with a lower frequency (2.16%).

This difference may be related to the level of analysis employed, unit versus patient. One hypothesis is that patients with a greater number of central access devices often correspond to more complex clinical cases, with a greater number of intravenous medications administered simultaneously, which would increase the likelihood of incompatibility occurrence regardless of the unit of admission.

The acceptance rate found in this study (81.8%) exceeded the 66.2% reported by Marsilio et al., who identified incompatibilities in ICU prescriptions and issued preparation and administration guidance.^10^ Although the comparison requires caution, given differences in the units of analysis and acceptance criteria, the good adherence reinforces the feasibility of pharmacist interventions at the time of administration. Cheon et al.^18^ reported a similar rate (90.4%) after implementing pharmacist monitoring of intravenous compatibility before dispensing in an ICU and a hemato-oncology ward in South Korea.

This strong adherence may relate to a gap observed by Machotka et al.,^17^ who noted that the nursing team, in practice, did not know how to resolve incompatibility problems, ascribing this difficulty to restricted access to compatibility data for the medications administered. The presence of the clinical pharmacist at the bedside appears to fill precisely this gap.

This overall acceptance rate, however, was not uniform across the different types of recommended action. Interventions with lower operational complexity, such as access flushing combined with staggered administration times, were accepted in their entirety (100%), whereas changing the infusion line was accepted in just over half of the cases (51.1%), and placement of a new venous access, the most invasive and logistically demanding of the three approaches, was accepted only 12.5% of the time. This gradient suggests that acceptance of pharmacist recommendations is related, at least in part, to the operational complexity and degree of invasiveness of the proposed action, rather than necessarily to the clinical severity of the identified incompatibility, reinforcing the importance of prioritizing less disruptive management strategies whenever feasible.

An even more striking result was reported in a prospective study conducted by a clinical pharmacist in an adult ICU, in which possible incompatibilities were identified through daily prescription review before administration, rather than through direct bedside observation as in the present study. In that context, the healthcare team accepted and implemented all 567 (100%) recommendations.^19^

The three approaches adopted, flushing with a compatible solution combined with staggered timing, changing the line, and recommending a new access, are aligned with strategies already described in the literature to reduce contact between incompatible medications. The choice between them, however, is not automatic: it depends on the infusion modality, the therapeutic urgency, lumen availability, the compatibility of the available flushing solutions, and any clinical restrictions of the patient.4., 5.

Other strategies include adjusting the mode of administration, prioritizing intermittent infusions when feasible, reviewing dosing schedules, converting from intravenous to oral administration, and substituting pharmacologically equivalent and compatible medications.^12^ The choice between them should consider the risk versus benefit relationship, weighing the expected clinical benefits versus risks such as reduced therapeutic efficacy, mechanical obstruction of infusion devices, and thrombosis.^12^

The considerable occurrence of incompatible drug pairs administered via the same line may reflect multiple factors related to the management of the problem, including possible lack of interest or low prioritization of the issue by staff, knowledge gaps in identifying and preventing such situations, and the absence of clear strategies or effective tools to reduce their occurrence.^17^ These factors indicate the necessity of structured measures, such as cross-compatibility charts, optimized administration schedules, and electronic decision-support systems, to support safer and more consistent practices.17., 18.

It is important to note that these structural strategies do not necessarily depend on expanding the clinical pharmacy staff, which broadens their applicability in resource-constrained settings. Printed cross-compatibility charts posted at nursing stations, standardized protocols for line flushing and staggered administration times, electronic alert systems integrated into the medical record, and periodic staff training are examples of low-cost measures. All are feasible to implement even in hospitals with limited pharmacy staffing, allowing incompatibility risk to be mitigated without requiring the hiring of additional specialized clinical pharmacists.17., 18.

The predominance of occurrences involving intermittent medications is consistent with findings from another study, in which 68.8% of the incompatibilities identified involved two medications administered by bolus.^5^ This does not mean the intermittent modality carries greater intrinsic risk, since that study did not calculate rates relative to the total number of exposure opportunities in each modality. A plausible hypothesis is higher turnover of intermittent medications in shared lines, which still warrants specific evaluation.

The combination of dexmedetomidine and ketamine was the pair most frequently identified as incompatible. A previous study assessed the physical compatibility of 29 pediatric combinations using three distinct analytical methods and demonstrated that dexmedetomidine is incompatible with ketamine across all techniques.^20^ These findings reinforce that concurrent intravenous administration of these medications should not be performed.

The most frequently recommended approach was to flush the system with a compatible solution and avoid simultaneous administration.^21^ Flushing reduces contact between sequentially administered medications and maintains catheter patency,^4^ and its absence increases the risk of incompatibilities between infusions and injections given in sequence.^7^ This practice, however, is not free of risk: interrupting and resuming the infusion to enable it may cause instability in the medication's blood concentration.^7^ The volume used also needs to be accounted for in patients with fluid restriction, using a solution compatible with the medications involved.^12^

The second most frequent intervention was changing the infusion line, which depends on the availability and organization of existing access devices. Although 63.6% of patients had a triple-lumen catheter, this does not eliminate the requirement of careful line planning, since multiple continuous and intermittent medications may compete for the same limited number of available access points.4., 12. Machotka et al.^17^ described a relationship between lumen availability and incompatibility risk, finding a lower mean number of lumens per patient in the medical ICU (1.88) than in the surgical ICU (2.41) and associating the difference with variation in incompatibility rates between the units.

Placement of a new access was indicated in only 5.0% of occurrences, consistent with its function as a last-resort option, reserved for situations in which separation is clinically necessary and unachievable with existing access devices, since additional devices expose the patient to mechanical and infectious risks. Multi-lumen devices may broaden the possibilities for separating infusions, but their performance depends on the system configuration and appropriate line management.4., 22.

The high occurrence of incompatibilities shows a systemic problem, resulting from polypharmacy, shared lines, variability in preparation, and the absence of compatibility data for numerous pairs, a situation typical of ICUs.4., 11. Local protocols, updated charts, ongoing training and active pharmacist involvement can support more consistent decisions and reduce avoidable exposures.4., 11., 23. In pediatric and neonatal units, a comparative evaluation of eight decision-support tools revealed differences in accuracy, completeness, scope, and applicability, with shortcomings in all of them, leading to a recommendation for standardized operating procedures for assessing incompatibilities.^24^

This study has limitations. The convenience sample covered 21.2% of admissions during the period, with an estimated margin of error of 9.3 percentage points, as detailed in the Methods, limiting the accuracy of the frequency estimates and introducing potential selection bias. In addition, the study was conducted at a single hospital that combined neonatal, pediatric, and adult units, which limits the generalizability of the findings.

The specific reasons for non-acceptance of the 29 refused pharmacist interventions (18.2% of the 159 proposed) were not systematically recorded in the data collection instrument, which precludes a reliable characterization of these cases. Structured recording of the reasons for non-adherence to pharmacist recommendations, including aspects related to the availability of access routes and healthcare team routine, constitutes a gap to be addressed in future studies.

The cross-sectional design, with each patient observed at a single point in time, made it impossible to characterize the configuration of infusions throughout the entire admission. The study also did not assess clinical outcomes attributable to incompatibilities or the longitudinal effects of the interventions. The findings depict the observed frequency of exposure and the immediate acceptability of the recommendations, but do not allow the conclusion that the interventions reduced adverse events or improved clinical outcomes.

Finally, the results depend on the quality and completeness of the information available in the databases consulted. Pairs lacking compatibility data should not be interpreted as necessarily incompatible: they represent a zone of uncertainty that requires prudent clinical management and highlights an experimental knowledge gap. This limitation is not exclusive to this study: observational surveys in other ICUs have also noted a considerable proportion of combinations lacking adequate compatibility data in the sources habitually consulted.11., 25.

This study also did not assess the appropriateness of the preparation practices used for medications administered intravenously (for example, dilution, final concentration, and order of manipulation), which may influence the compatibility observed at the bedside and constitutes a relevant factor to be investigated in future studies.

The data collection form was developed in-house by the research team, based on the study's own design and the prior definition of the variables of interest; no equivalent, validated structured instrument specifically aimed at direct bedside observation of drug incompatibilities was identified in the literature consulted. This absence of a prior reference instrument constitutes an additional limitation of this study.

Relative to studies that are limited to estimating potential incompatibilities from prescriptions, medical records, or nursing schedules, the main contribution of this study is to confirm, through direct bedside observation, the patient's actual exposure to the concomitant administration of incompatible medications, taking into account the effective configuration of venous access and the real simultaneity of administration, rather than only their coexistence in the prescription.

## Conclusion

By directly and prospectively assessing intravenous administration at the bedside, this study demonstrates that critically ill patients' real exposure to incompatible drug combinations, or to combinations lacking compatibility data, is frequent and that the clinical pharmacist's intervention at that time is accepted by the healthcare team in most identified situations. This approach supplements screening based solely on prescriptions by incorporating the actual venous access configuration and confirming the route of administration at the bedside.

The results support the functional feasibility of integrating pharmacists into infusion assessment and may guide the development of local protocols for this purpose. Future studies with greater sample coverage, longitudinal follow-up and assessment of clinical outcomes are still needed to determine the effect of these strategies on patient safety.

## Declaration of generative AI and AI-assisted technologies in the manuscript preparation process

During the preparation of this work, the corresponding author used Claude (Anthropic) and Perplexity to support the clarity and linguistic quality of the manuscript. After using these tools, the author(s) reviewed and edited the content as needed and take full responsibility for the content of the published article.

## Previous presentation

This manuscript has not been presented previously and is not under consideration for publication elsewhere.

## CRediT authorship contribution statement

**Allan Carneiro de Souza:** Writing – review & editing, Writing – original draft, Methodology, Investigation, Conceptualization. **Joiciane Dias Rodrigues Neves:** Writing – review & editing, Investigation. **André Luis Félix da Silva Terres:** Writing – original draft. **Gabriel Gonçalves da Silva:** Writing – original draft, Investigation. **Luana Ferreira de Almeida:** Writing – review & editing, Supervision. **Arnaldo Couto:** Writing – review & editing, Supervision. **Sabrina Calil Elias:** Writing – review & editing, Supervision. **Selma Rodrigues de Castilho:** Writing – review & editing, Supervision.

## Ethics approval

The project was approved by the institution's Research Ethics Committee (CAAE No. 83646024.0.0000.5259; Approval No. 7.175.924), with a waiver of informed consent, given that the pharmacist interventions evaluated were part of routine clinical practice and the study was observational.

## Funding

This research did not receive any specific grant from funding agencies in the public, commercial, or not-for-profit sectors.

## Declaration of competing interest

The authors declare that they have no known competing financial interests or personal relationships that could have appeared to influence the work reported in this paper.

## Appendix A Supplementary data

Supplementary material 1 Supplementary Table S1, presenting the 48 drug combinations identified in a single occurrence (*n* = 1), with the classification according to Micromedex and according to ASHP Injectable Drug Information and the final classification for each, is provided as a separate file (Supplementary_Table_S1.docx).

Supplementary material 2 STROBE checklist for cross-sectional studies.
