# Supplementary material for: Bedside pharmacist interventions for managing intravenous drug incompatibilities in critically ill patients
Source: Explor Res Clin Soc Pharm. 2026 Oct 15;24:100863. doi: 10.1016/j.rcsop.2026.100863 (PMC13717157; doi:10.1016/j.rcsop.2026.100863)
Supplement: Supplementary file 1 [file mmc1.docx]

**Supplementary Table S1**

**Supplementary Table S1. Combinations of incompatible drugs or drugs lacking compatibility data identified in a single occurrence (n = 1; 48 combinations; 48 of 159 total occurrences), complementary to Table 2.**

| **Drug pair** | **Micromedex Classification** | **n (%)** | **Micromedex Type** | **ASHP Classification** | **ASHP Type** | **Final classification** |
| --- | --- | --- | --- | --- | --- | --- |
| Acyclovir x aztreonam | Incompatible | 1 (0.6) | Physical (precipitation) | Incompatible | Physical (precipitation) | **Incompatible** |
| Albumin x vancomycin | Incompatible | 1 (0.6) | Physical (precipitation) | Incompatible | Physical (precipitation) | **Incompatible** |
| Ampicillin and sulbactam x ondansetron | Incompatible | 1 (0.6) | Physical (precipitation) | Incompatible | Physical (precipitation) | **Incompatible** |
| Ampicillin and sulbactam x polymyxin B ᵃ | Variable | 1 (0.6) | - | Not tested | - | **Incompatible** |
| Ampicillin and sulbactam x amphotericin B | Incompatible | 1 (0.6) | Physical (precipitation) | Not tested | - | **Incompatible** |
| Amphotericin B x linezolid | Incompatible | 1 (0.6) | Physical (precipitation) | Incompatible | Physical (precipitation) | **Incompatible** |
| Amphotericin B x meropenem | Incompatible | 1 (0.6) | Physical (precipitation) | Incompatible | Physical (precipitation) | **Incompatible** |
| Amphotericin B x metoclopramide | Incompatible | 1 (0.6) | Physical (precipitation) | Not tested | - | **Incompatible** |
| Amphotericin B x polymyxin B | Incompatible | 1 (0.6) | Physical (precipitation) | Not tested | - | **Incompatible** |
| Liposomal amphotericin B x meropenem ᵇ | Variable | 1 (0.6) | Not determined | Compatible | - | **Incompatible** |
| Sodium bicarbonate x thiamine | Incompatible | 1 (0.6) | Chemical (decomposition) | Not tested | - | **Incompatible** |
| Cefepime x omeprazole | Not tested | 1 (0.6) | - | Not tested | - | **Not tested** |
| Cefepime x magnesium sulfate | Incompatible | 1 (0.6) | Physical (opacity) | Not tested | - | **Incompatible** |
| Cefepime x teicoplanin | Not tested | 1 (0.6) | - | Not tested | - | **Not tested** |
| Ceftazidime x hydralazine | Incompatible | 1 (0.6) | Physical (turbidity/particles) | Not tested | - | **Incompatible** |
| Ceftazidime x nitroprusside | Incompatible | 1 (0.6) | Physical (turbidity/particles) | Not tested | - | **Incompatible** |
| Ketamine x meropenem | Incompatible | 1 (0.6) | Physical (precipitation) | Incompatible | Physical (precipitation) | **Incompatible** |
| Cisatracurium x ketamine | Incompatible | 1 (0.6) | Physical (particles) | Incompatible | Physical (precipitation) | **Incompatible** |
| Potassium chloride x sulfamethoxazole and trimethoprim | Incompatible | 1 (0.6) | Physical (turbidity/precipitation) | Not tested | - | **Incompatible** |
| Dexamethasone x sulfamethoxazole and trimethoprim | Incompatible | 1 (0.6) | Physical (turbidity/precipitation) | Not tested | - | **Incompatible** |
| Phenytoin x furosemide | Incompatible | 1 (0.6) | Physical (precipitation) | Not tested | - | **Incompatible** |
| Phenytoin x calcium gluconate | Incompatible | 1 (0.6) | Physical (precipitation) | Not tested | - | **Incompatible** |
| Phenytoin x hydralazine | Incompatible | 1 (0.6) | Physical (precipitation) | Not tested | - | **Incompatible** |
| Phenytoin x polymyxin B | Incompatible | 1 (0.6) | Physical (precipitation) | Not tested | - | **Incompatible** |
| Phenytoin x vancomycin | Incompatible | 1 (0.6) | Physical (precipitation) | Incompatible | Physical (precipitation) | **Incompatible** |
| Phenobarbital x omeprazole | Not tested | 1 (0.6) | - | Not tested | - | **Not tested** |
| Phenobarbital x ondansetron | Incompatible | 1 (0.6) | Physical (turbidity, particle, color change) | Not tested | - | **Incompatible** |
| Fluconazole x omeprazole | Not tested | 1 (0.6) | - | Not tested | - | **Not tested** |
| Fluconazole x paracetamol | Not tested | 1 (0.6) | - | Not tested | - | **Not tested** |
| Fluconazole x teicoplanin | Not tested | 1 (0.6) | - | Not tested | - | **Not tested** |
| Potassium phosphate x calcium gluconate | Incompatible | 1 (0.6) | Physical (precipitation) | Variable (concentration-dependent) | Not determined | **Incompatible** |
| Furosemide x nalbuphine | Incompatible | 1 (0.6) | Physical (turbidity, particle, color change) | Not tested | - | **Incompatible** |
| Furosemide x omeprazole | Not tested | 1 (0.6) | - | Not tested | - | **Not tested** |
| Furosemide x sulfamethoxazole and trimethoprim | Incompatible | 1 (0.6) | Physical (turbidity/precipitation) | Not tested | - | **Incompatible** |
| Furosemide x teicoplanin | Not tested | 1 (0.6) | - | Not tested | - | **Not tested** |
| Calcium gluconate x oxacillin | Incompatible | 1 (0.6) | Physical (turbidity, particle, color change) | Not tested | - | **Incompatible** |
| Calcium gluconate x sulfamethoxazole and trimethoprim | Incompatible | 1 (0.6) | Physical (turbidity/precipitation) | Not tested | - | **Incompatible** |
| Hydralazine x nitroprusside | Incompatible | 1 (0.6) | Physical (turbidity, particle, color change) | Not tested | - | **Incompatible** |
| Insulin lispro x 50% glucose | Not tested | 1 (0.6) | - | Not tested | - | **Not tested** |
| Insulin lispro x meropenem | Not tested | 1 (0.6) | - | Not tested | - | **Not tested** |
| Insulin lispro x vasopressin | Not tested | 1 (0.6) | - | Not tested | - | **Not tested** |
| Methylprednisolone x sulfamethoxazole and trimethoprim | Not tested | 1 (0.6) | - | Not tested | - | **Not tested** |
| Omeprazole x piperacillin and tazobactam | Not tested | 1 (0.6) | - | Not tested | - | **Not tested** |
| Omeprazole x sodium bicarbonate | Not tested | 1 (0.6) | - | Not tested | - | **Not tested** |
| Omeprazole x teicoplanin | Not tested | 1 (0.6) | - | Not tested | - | **Not tested** |
| Ondansetron x rituximab | Incompatible | 1 (0.6) | Physical (microprecipitation) | Not tested | - | **Incompatible** |
| Sulfamethoxazole and trimethoprim x vancomycin | Incompatible | 1 (0.6) | Physical (turbidity/precipitation) | Not tested | - | **Incompatible** |
| Liposomal amphotericin B x polymyxin B | Not tested | 1 (0.6) | - | Not tested | - | **Not tested** |

*ᵃ Ampicillin and sulbactam x polymyxin B: variable classification in Micromedex; considered incompatible because, under the conditions of this study, polymyxin B was diluted in glucose solution, in which ampicillin/sulbactam is unstable.*

*ᵇ Liposomal amphotericin B x meropenem: conflicting results in Micromedex (one study compatible, one incompatible); considered incompatible under the administration conditions observed in this study.*
