# Supplementary material for: Bedside pharmacist interventions for managing intravenous drug incompatibilities in critically ill patients
Source: Explor Res Clin Soc Pharm. 2026 Oct 15;24:100863. doi: 10.1016/j.rcsop.2026.100863 (PMC13717157; doi:10.1016/j.rcsop.2026.100863)
Supplement: Supplementary file 2 [file mmc2.docx]

**STROBE Checklist: Cross-Sectional Studies (updated version)**

*Bedside Pharmacist Interventions for Managing Drug Incompatibilities in Critically Ill Patients*

*Page numbering updated to match the revised version of the manuscript (Clean version, 18 pages, including references). Page numbers are approximate and may vary slightly after the journal's final typesetting.*

| **Section** | **Item** | **Recommendation** | **Page** |
| --- | --- | --- | --- |
| Title and abstract | 1 | (a) Indicate the study's design with a commonly used term in the title or the abstract | 1 |
|  |  | (b) Provide in the abstract an informative and balanced summary of what was done and what was found | 1-2 |
| **Introduction** |  |  |  |
| Background/rationale | 2 | Explain the scientific background and rationale for the investigation being reported | 2 |
| Objectives | 3 | State specific objectives, including any prespecified hypotheses | 3 |
| **Methods** |  |  |  |
| Study design | 4 | Present key elements of study design early in the paper | 3 |
| Setting | 5 | Describe the setting, locations, and relevant dates, including periods of recruitment, exposure, follow-up, and data collection | 3 |
| Participants | 6 | (a) Give the eligibility criteria, and the sources and methods of selection of participants | 3 |
| Variables | 7 | Clearly define all outcomes, exposures, predictors, potential confounders, and effect modifiers. Give diagnostic criteria, if applicable | 4-6 |
| Data sources/measurement | 8* | For each variable of interest, give sources of data and details of methods of assessment (measurement) | 4-6 |
| Bias | 9 | Describe any efforts to address potential sources of bias (evaluator training, pilot testing, and interobserver agreement check) | 4 |
| Study size | 10 | Explain how the study size was arrived at, including the post-hoc margin of error derivation | 3 |
| Quantitative variables | 11 | Explain how quantitative variables were handled in the analyses | 7-8 |
| Statistical methods | 12 | (a) Describe all statistical methods, including the chi-square and Mann-Whitney tests used for exploratory analyses | 7 |
|  |  | (c) Explain how missing data were addressed | 7 |
| **Results** |  |  |  |
| Participants | 13* | (a) Report numbers of individuals at each stage of the study; (c) consider use of a flow diagram | 8 |
| Descriptive data | 14* | (a) Give characteristics of study participants (demographic, clinical), including age and sex (Table 1) | 8 |
| Outcome data | 15* | Report numbers of outcome events or summary measures | 8-9 |
| Main results | 16 | (a) Give unadjusted estimates and results of the exploratory statistical tests (chi-square, Mann-Whitney) | 9 |
| **Discussion** |  |  |  |
| Key results | 18 | Summarise key results with reference to study objectives | 11 |
| Limitations | 19 | Discuss limitations of the study, including the 9.3 percentage-point margin of error associated with the convenience sample | 15 |
| Interpretation | 20 | Give a cautious overall interpretation of results, including comparison with Machotka et al. regarding lumens | 12, 14 |
| Generalisability | 21 | Discuss the generalisability (external validity) of the study results | 15 |
| **Other information** |  |  |  |
| Funding | 22 | Give the source of funding and the role of the funders | 16 |

**Give information separately for exposed and unexposed groups, when applicable.*

*Note: the journal-required "Key messages" section is reported on page 1 (Abstract/Highlights).*
